# The Efficacy of Fecal Microbiota Transplantation in Mouse Models Infected with *Clostridioides difficile* from the Perspective of Metabolic Profiling: A Systematic Review

**DOI:** 10.3390/metabo14120677

**Published:** 2024-12-03

**Authors:** Anna Voziki, Olga Deda, Melania Kachrimanidou

**Affiliations:** 1 Department of Microbiology, Medical School, Aristotle University of Thessaloniki, 54124 Thessaloniki, Greece; 2 Laboratory of Forensic Medicine & Toxicology, Department of Medicine, Aristotle University of Thessaloniki, 54124 Thessaloniki, Greece; 3 Biomic AUTh, Center for Interdisciplinary Research and Innovation (CIRI-AUTH), Balkan Center B1.4, 10th km Thessaloniki-Thermi Rd., 57001 Thessaloniki, Greece

**Keywords:** *Clostridioides difficile*, *Clostridioides difficile* infection (CDI), fecal microbiota transplantation (FMT), metabolomics, mouse models, gut microbiota, bile acids, short-chain fatty acids (SCFAs), mass spectrometry, metabolic profiling

## Abstract

**Objectives**: This systematic review evaluates the effectiveness of fecal microbiota transplantation (FMT) in treating *Clostridioides difficile* infection (CDI) in mouse models using a metabolomics-based approach. **Methods**: A comprehensive search was conducted in three databases (PubMed, Scopus, Google Scholar) from 10 April 2024 to 17 June 2024. Out of the 460 research studies reviewed and subjected to exclusion criteria, only 5 studies met all the inclusion criteria and were analyzed. **Results**: These studies consistently showed that FMT effectively restored gut microbiota and altered metabolic profiles, particularly increasing short-chain fatty acids (SCFAs) and secondary bile acids, which inhibited *C. difficile* growth. FMT proved superior to antibiotic and probiotic treatments in re-establishing a healthy gut microbiome, as evidenced by significant changes in the amino acid and carbohydrate levels. Despite its promise, variability in the outcomes—due to factors such as immune status, treatment protocols, and donor microbiome differences—underscores the need for standardization. Rather than pursuing immediate standardization, the documentation of factors such as donor and recipient microbiome profiles, preparation methods, and administration details could help identify optimal configurations for specific contexts and patient needs. In all the studies, FMT was successful in restoring the metabolic profile in mice. **Conclusions**: These findings align with the clinical data from CDI patients, suggesting that FMT holds potential as a therapeutic strategy for gut health restoration and CDI management. Further studies could pave the way for adoption in clinical practice.

## 1. Introduction

*Clostridioides difficile*, an anaerobic Gram-positive bacterium, exists in two distinct forms: vegetative cells and highly resistant spores [1]. The ability to form spores is critical for the persistence and recurrence of *C. difficile* infection (CDI), as these spores exhibit resistance to the hostile conditions within the gastrointestinal tract. The transmission of *C. difficile* spores predominantly occurs via the gastrointestinal route, facilitated mainly by contaminated food or inadequate hand hygiene. The clinical manifestations of CDI range from mild diarrhea to severe pseudomembranous colitis and toxic megacolon. Risk factors for CDI include advanced age (>65 years), prolonged hospitalization, comorbidities, and prior antibiotic exposure, all of which can weaken the immune defenses and disrupt the gut microbiota [1,2,3,4,5,6].

Antibiotics remain the primary treatment modality for CDI, although the choice of agents has evolved. Metronidazole, once a first-line therapy, is now less favored due to its lower clinical efficacy and higher recurrence rates. Current guidelines recommend oral vancomycin and fidaxomicin for the initial episode of CDI, with fidaxomicin being particularly advantageous due to its bactericidal properties and reduced impact on the gut microbiota, unlike the bacteriostatic effects of metronidazole and vancomycin. In response to rising concerns over antibiotic resistance, alternative therapies such as probiotics (e.g., *Saccharomyces boulardii*, *Lactobacillus plantarum*, *Bifidobacterium bifidum*) have been explored, showing promising results in preventing recurrent CDI (rCDI), although definitive evidence for their efficacy is still lacking. Other emerging treatments include bezlotoxumab, a monoclonal antibody targeting toxin B, bacteriophage therapies specific to *C. difficile*, and vaccine development [1,2,5,7,8,9,10].

Fecal microbiota transplantation (FMT) has emerged as a highly effective treatment for rCDI, particularly in patients with dysbiosis induced by antibiotic therapy. FMT aims to restore the gut microbiome, enhancing both alpha and beta diversity and increasing the abundance of microbial competitors against *C. difficile*. This approach has demonstrated cure rates between 85 and 91% [11]. Various methods for administering FMT exist, including oral capsules, nasojejunal tubes, and rectal delivery via colonoscopy or enema, with colonoscopy often considered the most effective due to its comprehensive delivery [5,11,12,13,14].

Metabolites, the intermediate molecules generated through various metabolic pathways, play crucial roles in energy production and biosynthesis in living organisms. The field of metabolomics, which studies these metabolites, can be divided into two main approaches: untargeted and targeted metabolomics. Untargeted metabolomics provide a broad survey of the metabolites present in a sample, exploring their relationships across different pathways, though they may not quantify all the metabolites precisely. In contrast, targeted metabolomics focus on specific groups of metabolites, allowing for precise quantification using standard curves. Mass spectrometry, often coupled with liquid or gas chromatography, is a key analytical technique in metabolomics, providing high sensitivity in identifying metabolites based on their mass-to-charge ratio (*m*/*z*) [15,16,17,18]. Gut microbiota significantly influences metabolic processes, and alterations in metabolite profiles can indicate disruptions due to disease or restoration following treatments like FMT.

Bile acids are a notable class of bioactive metabolites. Primary bile acids, synthesized from cholesterol in the liver and conjugated with taurine or glycine, are secreted into the intestine, where gut microbes convert them into secondary bile acids. An intact gut microbiome is essential for the regulation of these bile acids. Primary bile acids are known to promote *C. difficile* growth, underscoring their importance in the bacterium’s lifecycle [13,19,20]. Short-chain fatty acids (SCFAs), derived from dietary fibers metabolized by commensal bacteria, are associated with both tissue damage and pathogenicity [21,22]. Additionally, *C. difficile* utilizes amino acids through the Stickland pathway, as electron donors and recipients for generating ATP, further complicating the infection dynamics [23].

Given the distinct metabolite changes associated with *C. difficile* germination and microbiota disruption, these metabolic alterations can serve as biomarkers for CDI. This systematic review aims to assess the efficacy of FMT in mouse models of *C. difficile* infection, with a specific focus on associated metabolomic changes.

## 2. Materials and Methods

### 2.1. Aim of the Systematic Review

This systematic review aimed to address the following question: “What is the efficacy of fecal microbiota transplantation (FMT) in mouse models, infected with *Clostridioides difficile* (*C. difficile*), from the perspective of metabolic profiling”?

### 2.2. Study Design

The protocol for this systematic review adhered to the Preferred Reporting Items for Systematic Reviews and Meta-Analyses (PRISMA) guidelines [24]. Although a registration number is not provided, as the review focuses on animal studies outside the typical scope of registries like PROSPERO, we have ensured full compliance with PRISMA standards to enhance transparency and rigor.

### 2.3. Search Strategy and Sources

To validate the effectiveness of the search keywords, a preliminary search was conducted using Scopus. This step ensured that the publications identified in the preliminary search were also captured in the formal search and confirmed the absence of another systematic review addressing the same question. Following this, a comprehensive search was executed in PubMed, Scopus, and Google Scholar from 10 April 2024 to 17 June 2024. The keywords employed included ‘c. difficile’, ‘clostridioides difficile’, ‘cdi’, ‘clostridioides difficile-induced colitis’, ‘clostridium difficile-induced colitis’, ‘mice’, ‘mouse’, ‘fecal microbiota transplantation’, ‘fmt’, ‘metabolic profiling’, ‘metabolomics’, ‘metabolites’, ‘metabonomics’, ‘lipidomics’, ‘lipid profiling’, ‘bile acids’, and ‘amino acids’.

In PubMed, the search encompassed all the fields with an English language filter. In Scopus, the search was confined to Title–Abstract–Keywords with an English language filter. On Google Scholar, no filters or limitations were applied. Full search queries for each database are available in Supplementary Materials. The search yielded 12 studies from PubMed, 29 from Scopus, and 419 from Google Scholar.

### 2.4. Selection Criteria

A total of 460 studies from the three databases were reviewed by two reviewers. Initially, duplicate papers and non-English papers (from Google Scholar) were eliminated, resulting in 435 studies for screening. These studies were assessed for relevance to the research question based on their titles and abstracts. Review articles and non-original research papers were excluded. After this initial screening, 26 relevant studies were retrieved, with no exclusions due to inaccessible full texts. These 26 studies underwent a full-text review to assess their eligibility and extract their data.

The inclusion criteria for the systematic review required the studies to include the following:

1. Perform FMT treatment;
2. Utilize laboratory mice;
3. Infect the mice with *C. difficile*;
4. Validate the treatment efficacy through a metabolomics-based analysis;

Exclusion criteria were applied to the studies that performed the following:

- Utilized patients or cell cultures instead of mouse models;
- Did not employ FMT as a treatment;
- Did not use metabolomics for efficacy assessment;
- Did not infect mice with *C. difficile*;
- Conducted metabolomics prior to *C. difficile* infection;
- Were overviews or addendums.

Ultimately, only five studies met all the inclusion criteria and were incorporated into the systematic review (Table 1 and Table 2).

### 2.5. Data Extraction and Analysis

**Figure 1:**
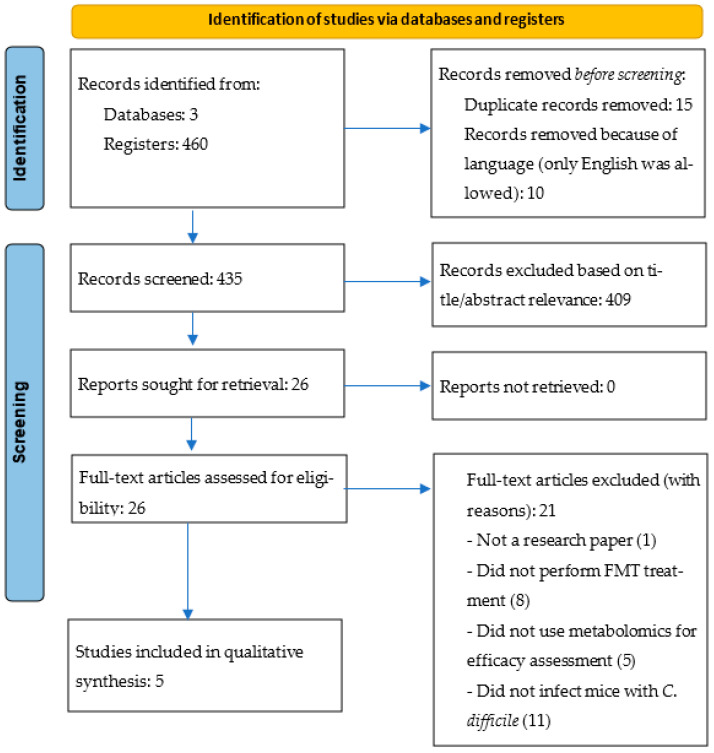
PRISMA flow chart for identification of studies via databases and registers.

Two independent reviewers meticulously extracted the pertinent data from each of the five studies, that met our predetermined criteria. We compiled comprehensive information from both the main manuscripts and their Supplementary Materials, focusing on details related to the animal models used, the experimental group assignments, and the administration of *C. difficile* and FMT, among other key experimental factors (Table 1). Additionally, we documented the biospecimens used, the sample preparation processes, and the metabolomics-based analytical techniques employed. We also identified the biomarkers associated with the FMT treatment of CDI in the animal models and the overall effects of FMT (Table 2). This aggregate flow chart (Figure 1) included crucial data on metabolite alterations and the impact of FMT on mice with *C. difficile* infections. We assessed bias using SYRCLE’s tool for animal studies. Most studies had a moderate risk of bias due to limited randomization and blinding, though controlled conditions helped reduce the risk in other areas. Overall, the risk was moderate, typical of preclinical studies. The effect measures were described qualitatively, focusing on the metabolic outcomes and comparisons between FMT and the control treatments.

## 3. Results

Following a comprehensive search in three major databases (PubMed, Scopus, and Google Scholar) using the specified keywords, a total of 460 studies were identified. After removing the duplicates and non-English studies, 435 papers remained for initial screening. Of these, 409 studies were excluded based on their title and abstract relevance, leaving 26 studies for full-text review. All the 26 studies were accessible. During the full-text review, 21 studies were excluded for the previously mentioned reasons. Consequently, five studies were included in our systematic review.

### 3.1. Animal/Mouse Models

A fundamental criterion in all the five studies was the use of laboratory mice. Most of the studies [25,26,27,28] employed the C57BL/6 mouse strain, whereas Battaglioli et al. (2018) [29] used germ-free Swiss Webster mice. Aggregate data revealed that the female mice were exclusively used in two studies [26,28]; Deda et al. (2022) [25] used only male mice, and Battaglioli et al. (2018) [29] included both sexes. According to Fachi et al. (2024) [30], female mice have an increased likelihood of recurrent *Clostridioides difficile* infection (CDI), which may explain their preference in certain models.

The C57BL/6 mouse strain is a preferred choice in CDI and FMT studies due to its well-documented genetic background, robust immune response, and broad availability, which collectively minimize variability in experimental outcomes. This strain’s genetic uniformity and well-characterized immune system make it ideal for studying infections and immunological therapies [31,32,33]. Conversely, Swiss Webster mice, being outbred, offer greater genetic diversity but also introduce more variability in results. The preference for C57BL/6 mice in CDI and FMT research is also reinforced by their susceptibility to a wide range of diseases and the reproducibility of experimental outcomes. While Swiss Webster mice are valuable in certain contexts due to their genetic variability, they can lead to more inconsistent outcomes in experimental settings [31,32,33].

Regarding the age of the mice, juvenile mice (less than 12 weeks old) were used in most of the studies [26,27,28,29], while Deda et al. (2022) [25] used adult mice aged 12 weeks. Fachi et al. (2024) [30] observed that the older infected mice (aged 7 to 14 months) exhibited more severe symptoms.

The number of mice used varied across the studies, ranging from 8 to 50 depending, also, on their experimental groups. Researchers must adhere to the “3Rs” principle (Reduce, Refine, Replace) to determine the minimum number of animals required for experiments due to ethical reasons and lower cost [34]. Two primary methods for calculating a sample size are a power analysis and the “resource equation” method, both of which rely on statistical tools [35]. Estimating statistical power and determining sample size are critical components of experimental design, especially in metabolomics research where identifying significant effects is essential. Despite their importance, these analyses are challenging and not commonly implemented due to several factors [36,37]. These include the necessity of pilot studies to estimate effect sizes and variability, the complex nature of metabolite data, and high biological variability. In the field of metabolic phenotyping, there is no standardized approach for this process, primarily because expected effects are often unknown in hypothesis-free research. The number, the type of analytes, and the effect sizes cannot be predicted in advance, making the process even more complicated.

### 3.2. Experimental Groups

In every study, mice were separated into different experimental groups and subjected to various treatments for comparative analysis. Deda et al. (2022) [25] separated 50 mice into five groups (G1–G5) to determine the efficacy of three different treatments used in clinical practice. Group G5 consisted of intact mice without any treatment or infection with *C. difficile*, serving as the control group. Group G4 consisted of mice that were infected but not treated. The other three groups (G1–G3) included mice infected with *C. difficile* and who were treated with metronidazole, probiotics, or FMT from the control mice for ten days. Additionally, the infected mice were exposed to an antibiotic cocktail for 4 days to disrupt their microbiome and make them more susceptible to CDI.

Xu et al. (2022) [26] split 32 mice into four groups: a control group without CDI and treatment (NC), a group with CDI but no treatment, and two groups infected with *C. difficile* and treated with either vancomycin or FMT, respectively, to compare the effects of these treatments. Similarly to the first study, all the groups except NC were exposed to antibiotics for five days before CDI.

Li et al. (2021) [28] separated 32 mice into four groups: a control group, a group (PBS group) exposed to antibiotics for five days, infected with *C. difficile* and treated with PBS for nine days, and two other groups exposed to antibiotics, infected with *C. difficile,* and treated with FMT or *B. thetaiotaomicron* (a probiotic) for nine days, to compare the probiotic and FMT treatments.

Littmann et al. (2021) [27] conducted two individual experiments to demonstrate the critical role of the immune system in supporting FMT. In the first experiment, four groups of mice were used: naïve intact mice as FMT donors and three groups of C57BL/6, C57BL/6 Rag1^Het^, and C57BL/6 Rag1^−/−^ mice. These mice were exposed to antibiotics for three days, infected with *C. difficile*, and treated with two doses of FMT. The second experiment involved three groups of mice: naïve intact mice, C57BL/6, and C57BL/6 C-II^−/−^ mice, treated similarly to those in the first experiment.

Battaglioli et al. (2018) [29] divided 12 germ-free mice into two groups: healthy-like mice (transplanted with stool from human-healthy-like mice) and dysbiotic mice (transplanted with stool from human–dysbiotic mice). Both of the groups received two doses of FMT prophylactically before *C. difficile* infection to assess the protective role of FMT.

The majority of studies prefer to utilize an antibiotic cocktail mouse model to explore CDI due to its better reflection of human CDI. However, germ-free mouse models are also important for studying CDI because of their non-antibiotic pretreatment advantage [38].

### 3.3. Clostridioides Difficile Infection in Mouse Models

Toxigenic strains of *C. difficile* (087 ribotype [VPI 10463], BI/NAP1/027 ribotype [CD186], and the 630 strain) were used in four out of the five experimental studies reviewed [26,27,28,29]. Only Deda et al. (2022) [25] utilized a non-toxigenic (tcdA^-^, tcdB^-^, cdtA^-^, cdtB^-^) *C. difficile* strain. This choice could be employed to differentiate between baseline microbiome shifts caused solely by bacterial colonization and those resulting from toxin-mediated disruptions, thereby enhancing our understanding of FMT’s effects under varying pathogenic conditions. This difference may be due to the varying immune responses to the toxigenic and non-toxigenic strains and the multiple antibiotic resistances to toxigenic strains, which did not align with their experiment design [33].

A minority of research teams preferred to infect mouse models with *C. difficile* spores [25,27] to ensure that the spore forms would not be eliminated by the immune system or flora of the mice, as spores are resilient and can survive in unfavorable environments [30,39]. However, Xu et al. (2022) [26], Li et al. (2021) [28], and Battaglioli et al. (2018) [29] chose to infect mice with the vegetative cells of *C. difficile*. This choice is supported by Castro-Córdova et al. (2020) [40], who claim that the heat treatment used to kill vegetative cells has many disadvantages in imitating a typical *C. difficile* infection. The infectious doses used were 10^7^ or 10^8^ CFU for the vegetative cells and 10^3^–10^4^ for the spores. The difference in infectious doses can be explained by the choice of using either vegetative or spore forms.

For spore cultures, Deda et al. (2022) [25] used Clospore liquid medium for 16 days under anaerobic conditions at 37 °C, followed by heating the culture to 65 °C to isolate the spores. For the vegetative cell cultures, Xu et al. [26] inoculated the *C. difficile* strain in BHI broth for 36 h at 37 °C under anaerobic conditions, while Battaglioli et al. (2018) [29] cultured the strain in both liquid and solid media for a minimum of 24 h at 37 °C under anaerobic conditions in an anaerobic chamber with a gas mixture (75% N_2_/20% CO_2_/5% H_2_). The remaining studies did not provide details about *C. difficile* culture or sporulation.

### 3.4. Experimental Key Factors

The mice were consistently provided with autoclaved ad libitum food and water [25,28], and Littmann et al. (2021) [27] maintained the mice under Specific Pathogen-Free (SPF) conditions, feeding them a grain-based diet that influences the alpha and beta diversity of the fecal microbiota, weight, metabolic profiling, and total health status [41]. Health metrics such as status, weight [25], survival rate, diarrhea [26], and the presence of *C. difficile* in the stool [27] were regularly monitored. The verification of *C. difficile* colonization was performed by culturing the homogenized fecal samples on TCCFA (taurocholate, cefoxitin, cycloserine, and fructose agar) plates [25] or a CDMN (*Clostridium difficile* Moxalactam Norfloxacin) agar medium [29].

Antibiotics were administered via drinking water in four studies [25,26,27,28] to disrupt the microbiome, effectively depleting the fecal microbiota within four days [42]. Deda et al. (2022) [25], Xu et al. (2022) [26], and Li et al. (2021) [28] employed an antibiotic cocktail containing metronidazole, vancomycin, kanamycin, gentamycin, and colistin, following the protocol by Chen et al. (2008) [43]. Littmann et al. (2021) [27] used a cocktail comprising neomycin, metronidazole, and vancomycin. Battaglioli et al. (2018) [29] chose transplantation with a dysbiotic microbiome to the germ-free mice instead of inducing gut dysbiosis with antibiotics, accurately reflecting the diversity and composition of human gut dysbiosis. Additionally, clindamycin was administered intraperitoneally to disrupt the anaerobic microflora in the studies involving antibiotic exposure [43].

*C. difficile* infection was typically induced via oral gavage [44], though Deda et al. (2022) [25] administered *C. difficile* spores through drinking water to avoid the stress or potential mortality associated with oral gavage [45]. In the study by VanInsberghe et al. [46], *C. difficile* spores were administered via drinking water, using a non-toxinogenic strain (ATCC BAA1801) at a concentration of 10^4^ spores/mL, starting one day before and continuing for two days after laxative treatment to simulate gastrointestinal disturbances and assess their impact on *C. difficile* colonization and recurrence.

The choice between gavage and administration via drinking water for substance delivery in mice depends on various factors, including the substance’s nature, the study design, and the desired outcomes. Gavage offers precision and control over dosage and timing, ensuring consistent delivery across subjects, even for substances with unpleasant taste or smell. However, gavage can stress subjects and may cause harm if not performed correctly, requiring highly trained lab personnel. Repeated gavage can lead to esophageal injury or other complications, depending on the type of gavage tube used, even when performed by experts. Conversely, administration via drinking water is less stressful and allows for prolonged administration, though it can result in inconsistent dosage, potential reduced intake, and dilution or stability issues if the water is not changed frequently [47,48].

Treatments (FMT, antibiotics, probiotics) were primarily administered via oral gavage for its efficiency and direct delivery to the stomach [49,50]. Littmann et al. (2021) [27] also used intrarectal instillation for the FMT doses. According to Mingaila et al. (2023) [51], intrarectal injection at lower dosages can more effectively restore microbiota with higher alpha diversity than FMT via oral gavage, as the inoculum does not need to navigate gastrointestinal barriers.

All the studies detailed their FMT preparation methods, mixing fecal samples or pellets with phosphate-buffered saline (PBS). Deda et al. (2022) [25] mixed 400 mg of fecal samples from control mice with 1 mL of sterile PBS. Xu et al. [26] used fresh feces from eight control mice diluted 1:10 in PBS. Li et al. (2021) [28] homogenized six fecal pellets from intact SPF mice with 3 mL PBS. Battaglioli et al. (2018) [29] combined six fecal pellets from mice previously transplanted with healthy human stool with 600 μL PBS. Littmann et al. (2021) [27] mixed 0.2 g of fresh fecal pellets from donor mice with 1 mL of deoxygenated PBS under anaerobic conditions. Proper homogenization of the fecal mixture via centrifugation or vortexing is crucial [50].

Regarding FMT doses, no significant variations were noted among the five studies. Deda et al. (2022) [25] administered 10% fecal water containing 400 mg/mL of fecal matter diluted in sterile PBS. Xu et al. (2022) [26] and Li et al. (2021) [28] administered 200 μL of the mixture, Littmann et al. (2021) [27] used 200 μL and 100 μL for two FMT doses, and Battaglioli et al. (2018) [29] administered 300 μL to each mouse. According to Bokoliya et al. (2021) [50], there is no ideal fecal infusion dose, with amounts typically ranging from 100 μL to 400 μL per dose. FMT administration is recommended from a single dose to twice per week for several weeks. A single dosage is not preferred due to the potential instability of the transplanted microbiome, while two doses per week can disrupt engraftment and weaken the newly established microbial community [50].

### 3.5. Metabolomics-Based Technique and Sample Preparation

The primary biospecimen used across the studies was fecal samples, which serve as a crucial medium for understanding the gut microbiota’s composition and its metabolic effects on host metabolism. The consistent use of fecal samples underscores their importance in capturing the metabolic changes associated with FMT and the gut’s response to *C. difficile* infection. Although Deda et al. (2022) [25] examined brain tissue, the fecal samples remained the predominant focus due to their direct relevance to gut health and microbial activity. The use of brain tissue served to explore the gut–brain axis and how FMT in a *C. difficile* mouse model could affect the brain metabolome through that philosophy.

The studies utilized a variety of sophisticated analytical techniques, each selected based on the specific metabolites or metabolic pathways of interest. Gas chromatography–mass spectrometry (GC-MS) and liquid chromatography–mass spectrometry (LC-MS) are usually employed [52]. GC-MS, in both its targeted and untargeted forms, is particularly valued for its ability to analyze volatile compounds and those amenable to derivatization. This technique was notably used in the studies by Deda et al. (2022) [25] and Xu et al. (2022) [26], where it provided detailed profiles of the organic acids and other metabolites.

LC-MS, another prevalent technique and sometimes considered the gold standard in metabolomics-based analyses [52,53], was used in these studies for detecting and quantifying the bile acids and other hydrophilic and lipophilic metabolites. This method’s high sensitivity and specificity make it ideal for comprehensive metabolic profiling. For instance, Li et al. (2021) [28] utilized LC-MS to measure the concentration of the various bile acids, highlighting its utility in detecting subtle metabolic shifts. Additionally, nuclear magnetic resonance (NMR) spectroscopy was employed in studies such as Battaglioli et al.’s (2018) [29], for targeted metabolomics, particularly in quantifying the amino acids. NMR spectroscopy’s non-destructive nature and ability to provide detailed structural information complemented the mass spectrometry techniques, offering a broader view of the metabolic landscape. The strength of NMR spectroscopy lies in its ability to measure a wide range of metabolites, but its disadvantage is its lower sensitivity compared to LC-MS, while GC-MS excels in volatile compound analysis. Apart from MS, which is a valuable tool in metabolomics-based analysis, other detectors like photodiode array (PDA) detectors are also used for targeted methods. For example, in Littmann et al. (2021) [27], PDA detectors were used for the analysis of bile acids, amino acids, and SCFAs.

Sample preparation methods vary significantly, reflecting the diversity of analytical techniques and the specific metabolites targeted. Common procedures include homogenization, extraction, centrifugation, and derivatization for specific metabolites. In the case of LC-MS, sample preparation often involves the use of organic solvents like methanol to extract the metabolites, as seen in the study by Littmann et al. (2021) [27]. The samples are typically centrifuged to remove particulates and then dried or reconstituted as needed to facilitate accurate measurement of the analytes.

When comparing the sample preparation methods for fecal samples across studies, it is evident that there is a degree of heterogeneity. This variability is primarily due to the different analytical techniques and metabolites targeted in each study. There are differences in the homogenization steps, extraction solvents, and the ratio of the solvents used. As reviewed by Deda et al. (2015) [52], the sample preparation for metabolic profiling using three analytical techniques—LC-MS, GC-MS and NMR spectroscopy—aims to extract as many metabolites as possible for untargeted profiling. The fewer the steps, the better, as fewer steps mean fewer errors. The concept of minimal sample preparation is optimal. Each analytical technique—whether GC-MS, LC-MS, or NMR spectroscopy—has unique requirements for sample cleanliness, solvent compatibility, and derivatization needs, which in turn influence the choice of sample preparation protocol [52,53].

### 3.6. Metabolomic Analysis and Effect of FMT

Following the administration of therapies (FMT, antibiotics, or probiotics) in each study included in our systematic review, research teams conducted metabolomic analyses to determine the metabolic alterations induced by these treatments. Specifically, Deda and colleagues [25] identified 217 metabolites, noting that 91 of these were significantly altered between the infected and untreated mice (G4) and the intact mice (G5). These included amino acids, nicotinic acid, pyridoxine, riboflavin, xanthine, glycerin, γ-aminobutyric acid, pyroglutamic acid, histamine, tryptamine, methylamine, trimethylamine, and taurine. Comparing the treated groups (G1, G2, G3) to G4 and G5, distinct alterations in the metabolites were observed, with the most changes seen in the metronidazole group (G1) (60 of 217 and 96 of 217, respectively). FMT therapy induced a metabolic profile more like the control group (G5), showing fewer changed metabolites (53 of 217) compared to metronidazole or the probiotics. It appears that while the metabolites such as the amino acids, especially glutamine, were increased in the groups treated with metronidazole, the probiotics or FMT compared to the infected and untreated mice (G4) and about 70% of the metabolites were decreased compared to the control mice. In the three treated groups, the serine, glycine (a co-germinant for *C. difficile* along with taurocholic acid [54]), arginine, inosine, and nicotinic acid decreased compared to the control group. The metronidazole and probiotic treatments increased the monoacylglycerol, 1-monooleoylglycerol, 3-hydroxybutyric acid, 4-hydroxybenzoic acid, acetylcarnitine, alanine, asparagine, and creatine, while FMT treatment increased the 2-ketoglutaric acid, 4-hydroxybenzoic acid, and 5-hydroxyindole-3-acetic acid. Additionally, a reversed trend was observed in each treated group. The amino acids, such as glutamine, xanthine, creatine and methylamine showed a reversed trend in all the treatments; the docosapentaenoic and eicosatrienoic acids showed a reversed trend with metronidazole and probiotics treatment; and arginine, betaine, choline, glutamine and glycine with FMT treatment. The N-acetyl-aspartate and lipophosphoglycans were altered only by FMT. Notably, the mice treated with metronidazole and probiotics exhibited more similar metabolic profiles compared to those treated with FMT, although none of these therapies fully restored the metabolic profile to the control state. Thus, FMT was more successful than the antibiotics or probiotics due to a higher number of reversed metabolites and distinct metabolic profiling in the brain.

In the study by Xu et al. (2022) [26], 125 metabolites were quantified, with 55 significantly altered between the infected and untreated mice and the uninfected and untreated mice. In the post-vancomycin treatment, more metabolites were altered (47 of 125) compared to the FMT treatment (26 of 125), suggesting that FMT results in a metabolic profile more like the control group. The vancomycin treatment increased the amino acids (tryptophan, glutamyl-valine, O-phosphoserine) and carbohydrates (raffinose, trehalose-6-phosphate, trisaccharides, sophorose, UDP–glucuronic acid) and decreased the erythritol, fructose-6-phosphate, maltotriose, sucrose, maltotriitol, and lactitol, which contribute to *C. difficile* germination and growth. Diets with high levels of carbohydrates, from any source, may have a protective role against *C. difficile*, regardless of *C. difficile* competitors’ reduction due to antibiotics [55]. Conversely, post-FMT treatment, all the mice survived with resolved inflammation, reduced levels of carbohydrates and amino acids, increased *Bacteroidetes* (not associated with dysbiotic microbiome and *C. difficile*-associated diarrhea [56]), and decreased Proteobacteria (a microbial signature of dysbiotic gut microbiota [57]). These microbiome alterations post-FMT resulted in a metabolic profile more like intact mice. *C. difficile* germination and growth were prevented due to restored carbohydrate and amino acid levels post-FMT, consistent with the high amino acid biosynthesis genes in healthy donor stool [58].

Li et al. (2021) [28] focused on bile acids, identifying 28 in total. In the infected mice who were administered PBS (PBS group), there was a decrease in alpha-muricholic acid, beta-muricholic acid, 12-ketolithocholic acid and deoxycholic acid, with a dramatic increase in taurocholic and glycodeoxycholic acid. In the FMT group, the highest levels of bile acids were observed, even higher than in the intact mice, with significant increases in alpha-muricholic acid, beta-muricholic acid, and deoxycholic acid. These alterations indicated that primary bile acids (excluding alpha- and beta-muricholic acids) promote *C. difficile* growth, while secondary bile acids (including alpha- and beta-muricholic acids) inhibit CDI [54]. Both the FMT and probiotic treatments decreased the ratio of promotion to inhibition of the bile acids, indicating less enhancement of *C. difficile* germination and growth, with significantly fewer copies of *C. difficile*, especially after FMT. FMT was more effective than probiotics, restoring the microbiome and reversing secondary bile acid composition, thereby restoring colonization resistance against *C. difficile* in the mouse models and humans [59].

Littmann et al. (2021) [27] assessed the FMT efficacy in immune-compromised mouse models: T and B cell-deficient Rag1^−/−^ mice, Rag1 heterozygous mice (Rag1^HET^), and MHC Class II^−/−^ (C-II^−/−^) mice, and metabolomic analysis-quantified amino acids, SCFAs, and bile acids. Before FMT, there was no metabolic alteration between the Rag1^−/−^ and Rag1^HET^ mice, but the metabolic profiles significantly shifted among the naïve and *C. difficile*-infected mice. Post-FMT, the Rag1^−/−^ mice did not restore their metabolite composition, with elevated primary bile acids and nearly undetectable secondary bile acids. In contrast, the Rag1^HET^ mice restored their metabolite profile to near-naïve levels, with enriched secondary bile acids (deoxycholic, lithocholic, taurodeoxycholic, and omega-muricholic). Comparing C-II^−/−^ mice to C57BL/6 mice, the deoxycholic and lithocholic acids were reduced post-FMT. Thus, FMT succeeded in the Rag1^HET^ mice, but failed in the Rag1^−/−^ and C-II^−/−^ mice, highlighting the importance of the host immune status through anti-inflammatory cytokines production in FMT success [56].

Battaglioli et al. (2018) [29] used germ-free mice transplanted with healthy or dysbiotic microbiomes and administered prophylactic FMT prior to *C. difficile* infection to assess the FMT efficacy. FMT in dysbiotic mice increased the SCFAs and secondary bile acids and reduced the free proline in the fecal samples. The SCFAs, such as butyrate, acetate, propanoate, valerate, as well as isovalerate and hexanoate were increased, consistent with the findings by Seekatz et al. (2018) [60], where butyrate, acetate, and propionate increased post-FMT in the patients. These metabolites play a vital role in enhancing gut barrier function and decreasing inflammation [61]. Butyrate correlates with *C. difficile* infection recovery, as it hampers *C. difficile* growth [62]. The secondary bile acids (deoxycholic, lithocholic, and ursodeoxycholic acids) increased the post-prophylactic FMT, while the primary bile acid taurocholic decreased, the cholic acid increased, and the chenodeoxycholic acid was undetectable post-FMT. In our opinion, a possible explanation for the undetectable levels of chenodeoxycholic acid could be that it is synthesized only in humans [59]. No SCFA or bile acid concentration changes were observed in the healthy mice post-FMT. Overall, prophylactic FMT increased the microbial richness, evenness and the resistance to *C. difficile*, and normalized the gut metabolic profiles to a healthier state.

Reviewing these five studies reveals similar metabolite tendencies in the mice before and after treatments. Amino acids decreased post-*C. difficile* infection without treatment, possibly due to consumption by metabolically active *C. difficile*, but in post-FMT or antibiotic treatment both increases and decreases could be observed [25,26,63]. The bile acids, categorized as primary or secondary, showed distinct patterns: the primary bile acids (excluding alpha- and beta-muricholic acids) remained elevated post-infection without treatment in the Rag1^−/−^ and C-II^−/−^ mice or dysbiotic mice pre-prophylactic FMT [27,28,29], while the secondary bile acids were significantly enriched post-bacteriotherapy, because the FMT restored bacteria that are able to produce bile salt hydrolase (BSH) and 7-α-dehydroxylase, enzymes for conversion from primary to secondary bile acids [61]. The amount of deoxycholic acid was consistently reduced pre-FMT or in the failed FMT cases (Rag1^−/−^, C-II^−/−^ mice) and increased post-successful FMT. Lithocholic acid increased post-prophylactic FMT in Battaglioli et al.’s (2018) study [29], but decreased in the immunodeficient C-II^−/−^ mice post-FMT in Littmann et al.’s (2021) study [27]. Due to the limited number of studies, common metabolite alterations are scarce for broader annotation.

## 4. Discussion

In this study, we aimed to assess the efficacy of fecal microbiota transplantation (FMT) in *Clostridioides difficile*-infected mouse models, with a focus on metabolomic analysis. Following a comprehensive search in three popular databases—PubMed, Scopus, and Google Scholar—using specific keywords, we identified five studies that met our inclusion criteria. The limited number of studies highlights the niche nature of this research area, as few research teams focus on using FMT in animal models with metabolomics to address CDI. Most excluded papers either did not use mouse models, did not implement FMT as a therapy, or lacked metabolomic analysis to assess the treatment efficacy.

We specifically selected studies involving animal models, as these are prevalent in biomedical research for preclinical testing and basic science investigations. While hamsters are commonly used in CDI research due to their susceptibility, they pose challenges such as a high risk of cross-contamination and a lack of model standardization. Mouse models are favored in FMT research due to their anatomical, physiological, and genetic similarities to humans, enabling controlled studies on gut microbiota alterations and consistent treatment administration. Unlike human studies, mouse models allow for precise control and the avoidance of complex comorbidities. While there is a reported ~90% similarity between human and mouse microbiomes at the phylum level, substantial differences in anatomy result in varying proportions and abundances of microbial phyla and genera, which in turn influence metabolite production [30,39,64,65,66]. Despite these taxonomic distinctions at finer levels, mouse models provide valuable, reproducible insights into microbial interactions and treatment outcomes in FMT studies.

Among the various mouse models, germ-free (gnotobiotic) mice and antibiotic-induced mice are commonly used in FMT experiments. Germ-free mice are characterized by the total absence of microbes in all their tissues and can be colonized with specific bacteria to mimic donor profiles. However, they are expensive, require specialized equipment and conditions, and may have limitations in their immune system, brain, and intestinal development and function. Conversely, antibiotic-induced mice are more cost-effective and do not require complete sterility, but they are not entirely free of microorganisms and may develop bacteria that is resistant to antibiotics [51,67,68].

The five studies we reviewed exhibited clear heterogeneity regarding the housing and breeding conditions of mice, as well as the FMT protocols used. Differences were noted in the amount of fecal samples and the volume of the FMT mixture administered. According to Secombe et al. (2021) [69], there is no standardized protocol or guidelines for FMT treatment in mouse models, as researchers often omit crucial experimental details. Variations were also observed in the amount and strain of the *C. difficile* administered, the preparation of the spores or colony-forming units (CFUs), and other specifics of the infection process. However, there were similarities in the administration methods, with most studies using oral gavage for treatments (FMT, probiotics, antibiotics) and *C. difficile* infection, due to the precise dose administration directly to the stomach. The antibiotic cocktails used to disrupt the gut microbiota were largely consistent, following the protocol by Chen et al. (2008) [43], which induced symptoms similar to those in patients.

Metabolomic analysis, an emerging tool in preclinical and clinical research, can be used for early diagnosis, treatment response prediction, and survival rate estimation by identifying prognostic biomarkers. Metabolites, the products of metabolic networks in living organisms, are crucial for cellular and tissue functions, energy production, signal transduction, and apoptosis. They include host-derived metabolites, those produced by microorganisms, and exogenous sources such as diet. Understanding metabolic mechanisms is essential for monitoring treatment development and addressing diseases [15,70,71]. The primary analytical techniques used in metabolomics are nuclear magnetic resonance (NMR) spectroscopy and mass spectrometry (MS), with the choice depending on the biospecimen and experimental goals. Metabolomic analysis has limitations, including the need for a specific experimental design and challenges in identifying metabolites, particularly with liquid chromatography–mass spectrometry (LC-MS) [15]. The five studies utilized GC-MS, LC-MS, and NMR spectrometric techniques to detect metabolites, primarily using fecal samples, which is a non-invasive method. Other biospecimens included brain and colon tissues and urine, with p-cresol sulfate serving as a biomarker for bacteriotherapy efficacy [72].

The studies consistently found metabolomic alterations in the mouse models post-FMT. The amino acids such as glutamine and glycine increased, while the free proline, arginine tryptophan, and valine decreased in the fecal samples. The carbohydrates generally showed a decrease. The short-chain fatty acids (SCFAs) such as butyrate, acetate, propionate, isovalerate, valerate, and hexanoate were restored post-FMT. The bile acids exhibited significant changes; the primary bile acids like taurocholic acid decreased, while the secondary bile acids and muricholic acids increased, inhibiting *C. difficile* germination. Metataxonomically, the *Bacteroidetes* increased while the Proteobacteria decreased.

McDonald et al. (2018) [73] observed similar patterns in patients, chemostat models, and batch cultures, where valerate (an SCFA) decreased while *C. difficile* spores increased. Post-FMT, the valerate and its precursors, such as 5-aminovalerate, ethanol, and propionate, increased, creating an environment unfavorable for *C. difficile*. Seekatz et al. (2018, 2015) [60,74] reported the full recovery of metabolites like butyrate, acetate, and propionate in six patients with recurrent CDI post-FMT. The role of butyrate in preventing *C. difficile* infection is attributed to its function in maintaining gut epithelial integrity and regulating immune responses. Lawley et al. (2012) [75] found that the SCFAs decreased, and succinate increased in the untreated *C. difficile*-infected mouse models due to succinate-producing *P. distasonis*. Kellogg et al. (2024) [76] corroborated this, noting that succinate accumulation in gut dysbiosis conditions activates immune responses in the colon. Nagao-Kitamoto et al. (2020) [77] found that pre-FMT treatment in the disrupted gut microbiomes of the mouse models led to succinate reduction, likely due to *Phascolarctobacterium* species restoration.

Our findings align with studies in CDI patients who received FMT, showing that primary bile acids, like taurocholic acid, promoting *C. difficile* growth were reduced post-FMT, while secondary bile acids, like deoxycholic, lithocholic, ursodeoxycholic, and others, increased [54,58,59,60,73,78]. The conversion of primary to secondary bile acids involves enzymes like bile salt hydrolase (BSH) and 7-α-dehydroxylase produced by healthy gut microbiota [54]. These metabolite changes correlated with increased *Lachnospiraceae* and *Ruminococcaceae* and reduced *Clostridiales* [59,60]. Weingarden et al. (2014) [78] noted that the patients’ gut microbiota, initially dominated by Proteobacteria, shifted post-FMT to a composition resembling the donor’s, with increased the *Bacteroidetes* and Firmicutes. McMillan’s et al.’s (2024) [58] experiments in CDI patients emphasized the changes in the lipid and amino acid levels in human feces. Specifically, the acylcarnitines were decreased post-FMT and amino acids, such as leucine, isoleucine, cysteine, proline, tryptophan, and valine, with a pivotal role in *C. difficile* growth as auxotrophies, were also decreased post-FMT. These alterations were correlated with metagenomic findings of the Proteobacteria phylum and *Enterobacteriaceae* family reduction. Alterations in the carbohydrates levels had lesser significance.

Our study has limitations, including the restriction to three databases due to access constraints and a focus on mouse model studies for preclinical FMT research. While numerous studies exist on human FMT treatments, our focus was on preclinical models. Sensitivity analyses were not performed due to the descriptive nature of this review and the limited number of studies. Regarding certainty, we did not use formal tools to assess it, which limits this review. Still, the consistent metabolic changes seen across the reviewed studies provide moderate confidence in the findings.

In our opinion, FMT appears to be a promising treatment for CDI, offering potential for gut microflora and metabolite restoration in both mice and humans, with better outcomes compared to antibiotic or probiotic treatments. According to Seekatz et al. (2015) [74], FMT provides long-term colonization resistance against *C. difficile*, aiding its clearance. However, concerns about FMT’s safety and efficacy remain, particularly due to the lack of standardized protocols and the risk of pathogen transfer from donor stool [79,80]. Further research is needed to better understand the metabolic alterations and assess FMT’s safety and efficacy in both animal models and humans.

## 5. Conclusions

This systematic review examined fecal microbiota transplantation (FMT) as a suggested treatment for *Clostridioides difficile* infection (CDI), particularly through its impact on gut metabolomics in mouse models. FMT consistently outperformed antibiotics and probiotics in restoring gut bacterial diversity and boosting beneficial metabolites, such as short-chain fatty acids (SCFAs) and secondary bile acids, which are essential for gut health and resistance against *C. difficile*. Despite these promising results, the development of standardized FMT protocols and further research into the metabolic mechanisms involved are crucial to translating these findings into human applications. Establishing uniform protocols, from selecting suitable donors to optimizing preparation and administration methods, is essential for ensuring consistent, safe outcomes. Human microbiomes are diverse, so tracking specific metabolic markers could help personalize FMT, allowing treatment to be tailored to individual patient needs. This approach could broaden FMT’s use beyond CDI, addressing wider metabolic and gut health issues. Applying these insights in clinical practice could close the gap between research and treatment, establishing FMT as a reliable, targeted therapy for infection control and overall gut health.

## Figures and Tables

**Table 1:** Aggregated table with extracted data, including the animal model used and their respective experimental groups, the *C. difficile* strain, and the experimental key factors from the 5 systematically included studies.

Reference	Animal Model	Experimental Groups	CD	Experimental Key Factors
Deda et al., 2022 [25]	50 C57BL/6 male mice, 12 weeks old 5 groups of 10 mice (G1–G5)	**G1**: antibiotic exposure (4 d), clindamycin injection, *C. difficile* infection, metronidazole (50 mg/kg/day) for 10 days. **G2**: antibiotic exposure (4 d), clindamycin injection, *C. difficile* infection, probiotics for 10 days. **G3**: antibiotic exposure (4 d), clindamycin injection, *C. difficile* infected, FMT (400 mg/mL) from controls for 10 days. **G4**: antibiotic exposure (4 d), clindamycin injection, *C. difficile* infected, no treatment. **G5**: intact.	Non-toxigenic (tcdA^-^ tcdB^-^ cdtA^-^ cdtB^-^) *C. difficile* strain cultured in Columbia blood agar plate (48 h, anaerobically, 37 °C) Clospore liquid medium culture (16 d, anaerobically, 37 °C). Only spores after heated (65 °C, 20 min). 10^4^ spores (2000 spores/20 μL, after dilution in sterile water).	Breeding: 12 h light/12 h dark cycle, temp. 22–25, 50% humidity, autoclaved ad libitum food and water, bedding. Recording health status/day, weight/week Verification of *C. difficile* colonization by culturing homogenized fecal samples on TCCFA plates. Brain tissue collection *postmortem*. Antibiotic cocktail, *C. difficile* infection and treatments via drinking water. Clindamycin via intraperitoneal injection. FMT: 10% fecal water (fecal samples of controls mice diluted in sterile PBS, 400 mg/mL).
Xu et al., 2022 [26]	32 C57BL/6 female mice 6–8 weeks old 4 groups of 8 mice (NC, CDI, Vanc, FMT)	**NC**: intact. **CDI**: antibiotic exposure (5 d), clindamycin injection, *C. difficile* infection. **Vanc**: antibiotic exposure (5 d), clindamycin injection, *C. difficile* infection, vancomycin for 5 d (50 mg/kg/d). **FMT**: antibiotic exposure (5 d), clindamycin injection, *C. difficile* infection, FMT.	*C. difficile* VPI 10,463. 10^8^ CFU of vegetative cells *C. difficile*. Inoculation in BHI broth (36 h, 37 °C, anaerobically).	Monitored daily diarrhea, weight change, survival rate. Antibiotic cocktail via drinking water, clindamycin via intraperitoneal injection, *C. difficile*, vancomycin, FMT by oral gavage. FMT: mixed fresh feces from 8 normal mice with PBS (1:10 dilution), 200 μL resuspended mixture via oral gavage at day 1.
Littmann et al., 2021 [27]	C57BL/6 mice 2–4 months old *1st experiment:* 4 groups, 21 mice (naive mice = 5, *C. difficile* infected = 6, *C. difficile* infected/FMT-treated Rag1^Het^ = 6, *C. difficile* infected/FMT-treated Rag1^−/−^ = 4), *2nd experiment:* 3 groups of total 8 mice (FMT-treated C57BL/6 = 3, *C. difficile* infected FMT-treated C-II^−/−^ = 5)	**Naive mice**: FMT donors, intact. *1st experiment* ***C. difficile* infected mice**: antibiotic exposure (for 72 h), clindamycin injection (200 μg, 48 h later), *C. difficile* infection (24 h later), 2 FMTs (200 μL + 100 μL and 200 μL). *C. difficile* infected/FMT-treated Rag1^Het^ mice: antibiotic exposure (for 72 h), clindamycin injection (200 μg, 48 h later), *C. difficile* infection (24 h later), 2 FMTs (200 μL + 100 μL and 200 μL). ***C. difficile* infected/FMT-treated Rag1^−/−^ mice**: antibiotic exposure (for 72 h), clindamycin injection (200 μg, 48 h later), *C. difficile* infection (24 h later), 2 FMTs (200 μL + 100 μL and 200 μL). *2nd experiment* ***C. difficile* infected FMT-treated C-II^−/−^ mice:** antibiotic exposure (for 72 h), clindamycin injection (200 μg, 48 h later), *C. difficile* infection (24 h later), 2 FMTs (200 μL + 100 μL and 200 μL). **FMT-treated C57BL/6 mice:** antibiotic exposure (for 72 h), clindamycin injection (200 μg, 48 h later), *C. difficile* infection (24 h later), 2 FMTs (200 μL + 100 μL and 200 μL).	*C. difficile* (CD196, ribotype 027 strain, and VPI10463, ribotype 087 strain). ~1000 spores received.	Breeding, sterile autoclaved cages SPF conditions, grain-based diet, autoclaved water ad libitum. Monitoring for *C. difficile* in feces and weight changes. Antibiotic exposure via drinking water, clindamycin via intraperitoneal injection, *C. difficile* infection via oral gavage. 1st doses FMT: flashing fecal pellets (at 0.2 g/mL) with deoxygenated PBS under anaerobic conditions, without food debris, via oral gavage (200 μL) and via intrarectal instillation (100 μL). 2nd dose via oral gavage (200 μL) after 24 h.
Li et al., 2021 [28]	32 C57BL/6 female mice, 6–7 weeks old, 17–19 g, 4 groups of 8 mice (control, PBS, FMT, probiotics).	**Control group:** intact. **PBS group:** antibiotic exposure (5 d), clindamycin intraperitoneal injection (20 mg/kg), *C. difficile* infection, PBS (200 μL) for 9 days. **FMT group:** antibiotic exposure (5 d), clindamycin intraperitoneal injection (20 mg/kg), *C. difficile* infection, FMT (200 μL) for 9 days. **Probiotics group:** antibiotic exposure (5 d), clindamycin intraperitoneal injection (20 mg/kg), *C. difficile* infection, PBS with 5 × 10^8^ CFU *B. thetaiotaomicron* (200 μL) for 9 days.	10^7^ CFUof toxigenic BI/NAP1/027 *C. difficile* strain.	Breeding (7 d): autoclaved bedding, food, water (with free access), under 12 h light cycle. Antibiotic exposure via drinking water, clindamycin via intraperitoneal injection, *C. difficile* and treatments via oral gavage. FMT: homogenization of 6 fecal pellets from untreated healthy SPF C57BL/6 mice in 3 mL PBS, centrifugation.
Battaglioli et al., 2018 [29]	12 female and male Germ-Free Swiss Webster mice, 4 weeks old, 2 groups of 6 mice (healthy-like mice, dysbiotic mice).	**Healthy-like mice:** transplanted GF mice with stool from healthy-like mice group, 4 w later 2 FMTs (300 μL, 4 d apart) from mice previously transplanted with stool from a healthy human donor, *C. difficile* infection (1 w post FMTs). **Dysbiotic mice:** transplanted GF mice with stool from dysbiotic mice group, 4 w later 2 FMTs (300 μL, 4 d apart) from mice previously transplanted with stool from a healthy human donor, *C. difficile* infection (1 w post FMTs).	~10^7^ CFU *C. difficile* strain 630. Cultured anaerobically in liquid and solid growth media for minimum 24 h at 37 °C, in anaerobic chamber with gas mixture (75% N_2_/20% CO_2_/5% H_2_).	Adaption of human-derived gut microbial communities to mice for 4 weeks after transplantation. 2 FMTs: 6 pellets and 600 μL PBS, vortexing, settling at 4 °C for up to 2 h. FMTs and *C. difficile* via oral gavage. Fecal samples collection before CDI and post on days 1, 2, 6. Verification of *C. difficile* colonization by culturing homogenized fecal pellets in CDMN agar medium, incubating anaerobically at 37 °C for 24 h.

**Table 2:** Aggregated table with extracted data, including the analytical technique, the biospecimen, the sample preparation for the metabolomic analysis, the discovered biomarkers, and the effect of FMT, from the 5 systematically included studies.

Reference	Analytical Technique	Biospecimen/Sample Preparation	Biomarkers	Effect of FMT
Deda et al., 2022 [25]	Untargeted GC-MS Targeted GC-MS/MS: for 52 organic acid metabolites Targeted HILIC-MS/MS: for 110 hydrophilic metabolites Untargeted RP-LC-HRMS/MS: for hydrophilic and lipophilic metabolic profiling	Brain tissues homogenization by a Bead Mill Homogenizer. **Hydrophilic Metabolites:** Tissues with MeOH: IPA H_2_O (1:1:2), in a ratio of tissue weight/solvent volume of 1:3 (*w*_br_/*v*_SOL_), vortexing, sonicating, centrifugation, and evaporating to dryness. Supernatant collection for HILIC-MS/MS and GC-MS. **Lipophilic Metabolites:** Tissues extraction with MTBE (3:1), in a ratio of tissue weight/solvent volume 1:3 (*w*br/*v*sol) vortexing, centrifugation, evaporating to dryness. **Derivatization** with 2% MeOX (70 °C, 2 h), followed by MSTFA with 1% TMCS (70 °C, 1 h). N-pentadecane as injection standard.	Total of 217 metabolites. Significantly altered metabolites: **G4-G5:** 91/217 decreased in G4 (amino acids, nicotinic acid, pyridoxine, riboflavin, xanthine, glycerin, *γ*-aminobutyric, pyroglutamic acids, histamine, tryptamine, methylamine, trimethylamine, taurine). **G1-G4:** 60/217. **G2-G4:** 48/217. **G3-G4:** 28/217. **G1-G5:** 96/217 (more different to the controls). **G2-G5:** 87/217. **G3-G5:** 53/217 (more similar to the controls). **G1-G2-G3:** increased metabolites compared to G4 (amino acids, glutamine), ~70% of metabolites decreased in G1/2/3 compared to G5 (serine, glycine, arginine, inosine, nicotinic acid). **G1-G2:** more similar response, increase of 1-Monoacylglycerol, 1-Monooleoylglycerol, 3-hydroxybutyric acid, 4-hydroxybenzoic acid, acetylcarnitine, alanine, asparagine, and creatine. **G3:** increase of 2-ketogloutaric acid, 4-hydroxybenzoic acid and 5-hydroxy indole-3-acetic acid. **Reversed trend in all treatment:** amino acids, glutamine, xanthine, creatine, methylamine. **Reversed trend in G1-G2:** docosapentaenoic, eicosatrienoic acids. **Reversed trend in G3:** arginine, betaine, choline, glutamine, glycine (in G1/G2 not as pronounced effect).	FMT with better results than the other treatments, more metabolites reversed, more distinct metabolic profiling. N-acetyl aspartate, and LPGs were altered only by FMT.
Xu et al., 2022 [26]	Untargeted GC-MS analysis	Fecal samples homogenization with ice-cold methanol, centrifugation. Supernatant with heptadecanoic acid, dried using a nitrogen stream. Derivatization with methoxamine (37 °C, 24 h) followed by BSTFA with 1% TMCS (70 °C, 2 h).	Total of 125 metabolites. Significantly differentiated metabolites: **NC-CDI:** 55 **NC-FMT:** 26 **NC-Vanc:** 47 **FMT-Vanc:** 48 **FMT-Vanc differences:** Vanc → increased amino acids (tryptophan, glutamyl-valine, O-phosphoserine), increased carbohydrates (raffinose, trehalose-6-phosphate, trisaccharides, sophorose, and UDP–glucuronic acid), decreased carbohydrates (erythritol, fructose-6-phosphate, maltotriose, sucrose, maltotriitol, and lactitol) → *C. difficile* germination and growth.	FMT metabolic profile closer to NC group. All 8 mice from FMT group survived. Resolution of inflammation. Increases of *Bacteroidetes* and decreases of Proteobacteria Metabolic differences between FMT-Vanc. Restoration of carbohydrates and amino acids for preventing *C. difficile* germination after FMT.
Littmann et al., 2021 [27]	**Bile acids quantification:** UPLC-MS **Amino acids quantification:** UPLC- Photodiode Detector Array **SCFAs quantification:** UPLC- Photodiode Detector Array	**Bile acids:** Suspension of fecal samples in methanol (5 μL/mg stool), vortexing, 2 cycles of centrifugation, and analysis. **Amino acids:** Homogenization of fecal samples in methanol (5 μL/mg stool), 2 cycles of centrifugation. Derivatization **SCFAs:** Homogenization of fecal samples in a volatile-free fatty acid mix (5 μL/mg stool), 2 cycles of centrifugation. Supernatant filtration through filter plates, and analysis.	**Prior FMT:** no significant difference between Rag1^Het^-Rag1^−/−^, difference between naive- *C. difficile* infected mice. **Post FMT:** Restoration of metabolic profile of Rag1^Het^ like naive mice, no restoration of Rag1^−/−^. Secondary bile acids (deoxycholic acid, lithocholic acid, taurodeoxycholic acid, and omega-muricholic acid) are significantly enriched in Rag1^Het^ post-FMT, in contrast with Rag1^−/−^. For Rag1^−/−^ mice, secondary bile acids are nearly undetectable, and primary bile acids remain elevated. Significantly reduced deoxycholic and lithocholic acid to FMT-treated C-II^−/−^ mice compared to FMT-treated C57BL/6 mice.	A failed FMT leads to functionally impairment of metabolites. FMT succession depends on host’s immune status. FMT non-responsive mice failed to restore 2° bile acid pools in the cecum. FMT successful for Rag1^Het^ mice, unsuccessful for Rag1^−/−^ mice. MHC Class II ^−/−^ (C-II^−/−^) mice failed to clear *C. difficile* post FMT.
Li et al., 2021 [28]	Bilie acids quantification: LC-MS	Feces collection and storation at −80 °C. Suspension of 100 mg of feces in 500 µL of a precipitant solution. Mixture centrifugation. Supernatant through a solid-phase extraction (SPE) column, volatilization by freeze-drying under vacuum, and dissolving the residue in 100 µL of 20% acetonitrile.	Total of 28 bile acids (BAs). **PBS group:** Significant decrease of alpha-muricholic acid, beta-muricholic acid, 12-ketolithocholic acid, and deoxycholic acid and dramatically increase of taurocholic acid and glycodeoxycholic acid. **FMT group:** the highest BAs levels -> alpha-muricholic acid, beta-muricholic acid, deoxycholic acid, (even higher than control group). **FMT and probiotic groups:** significantly decreased the ratio of promotion/inhibition BAs, no significant difference among FMT-probiotics-control groups. Promotion CDI: primary bile acids, excluded alpha-muricholic, beta-muricholic. Inhibition CDI: secondary bile acids, included alpha-muricholic, beta-muricholic.	Significantly less *C. difficile* copies after FMT and probiotic (FMT more effective). FMT reversed bile acids composition after CDI. Decreased ratio promotion/inhibition BAs after FMT that indicate FMT does not enhance the germination and growth of *C. difficile* and restores cecal microbiome and metabolic profile after CDI.
Battaglioli et al., 2018 [29]	NMR spectroscopy Targeted and untargeted analysis for SCFAs: GC-MS Secondary bile acids: LC-MS	**NMR spectroscopy** **for amino acids:** Resuspension: feces in 1 mL of molecular grade water. Freeze-Thaw Cycles: 3 rounds by dry ice. Homogenization by bead beating, centrifugation, and analysis of frozen supernatant. Vortexing thawed samples with 0.1 M phosphate buffer and 1 mM TSP-d_4_ solution in D_2_O, and analysis. **Untargeted metabolomics:** Extraction by Zirconia beads in acidified water and acetonitrile, and centrifugation. Resuspension in 5% formic acid/5% acetonitrile in water. **SCFAs:** Extraction: mixing frozen stool with acidified water containing 6 μg/mL sodium butyrate-(^13^C)_4_, in a 50 μL/mg ratio. Vortexing sonication, and, centrifugation. **Secondary bile acids:** Extraction: mixing stool with 0.1 M NaOH containing internal standards. Vortexing. Freezing samples overnight at −80 °C, centrifugation.	Decreased free proline after prophylactic FMTs in dysbiotic mice. Increased SCFAs (butyrate, acetate, propanoate, isovalerate, valerate),hexanoate) and secondary bile acids (deoxycholic, lithocholic, ursodeoxycholic acids) after prophylactic FMTs in dysbiotic mice. Primary bile acids: decreased taurocholic acid, increased cholic acid, undetectable chenodeoxycholic acid post prophylactic FMT in dysbiotic mice. No differences in SCFAs and secondary bile acids concentrations in healthy mice post prophylactic FMTs.	Significant increase in microbial richness and evenness post prophylactic FMTs in dysbiotic mice. Resistance to CDI after prophylactic FMTs in dysbiotic mice without detectable *C. difficile* in fecal samples. Significant decrease of free proline post prophylactic FMTs in dysbiotic mice. Normalization of the overall gut metabolic milieu to a healthier state. No detectable effect of prophylactic FMTs in healthy-like mice.

## Data Availability

No new data were created or analyzed in this study.

## Supplementary Materials

The following supporting information can be downloaded at https://www.mdpi.com/article/10.3390/metabo14120677/s1. With advanced search queries for PubMed, Scopus, Google Scholar databases.
