# Supplementary material for: The Efficacy of Fecal Microbiota Transplantation in Mouse Models Infected with Clostridioides difficile from the Perspective of Metabolic Profiling: A Systematic Review
Source: Metabolites. 2024 Dec 3;14(12):677. doi: 10.3390/metabo14120677 (PMC11679247; doi:10.3390/metabo14120677)
Supplement: Supplementary file 1 [file metabolites-14-00677-s001.zip › metabolites-3244389-supplementary.pdf]

Supplementary Information

The Efficacy of Fecal Microbiota Transplantation in Mouse Models Infected with *Clostridioides difficile* from the Perspective of Metabolic Profiling: A Systematic Review

Anna Voziki [1], Olga Deda [2,3,*] and Melania Kachrimanidou [1,*]

[1] Department of Microbiology, Medical School, Aristotle University of Thessaloniki, 54124 Thessaloniki, Greece

[2] Laboratory of Forensic Medicine & Toxicology, Department of Medicine, Aristotle University of Thessaloniki, 54124 Thessaloniki, Greece

[3] Biomic AUTh, Center for Interdisciplinary Research and Innovation (CIRI-AUTH), Balkan Center B1.4, 10th km Thessaloniki-Thermi Rd., 57001 Thessaloniki, Greece

*Correspondence: olgadeda@auth.gr (O.D.); melinaka@auth.gr (M.K.)

Supplementary Methods

PubMed advanced search query:

('c. difficile' OR 'clostridioides difficile' OR 'cdi' OR 'clostridioides difficile-induced colitis' OR 'clostridium difficile-induced colitis') AND ('mice' OR 'mouse') AND ('fecal microbiota transplantation' OR 'fmt') AND ('metabolic profiling' OR 'metabolomics' OR 'metabolites' OR 'metabonomics' OR 'lipidomics' OR 'lipid profiling' OR 'bile acids' OR 'amino acids')

Scopus advanced search query:

(TITLE-ABS-KEY (c.difficile) OR TITLE-ABS-KEY (clostridioides AND difficile) OR TITLE-ABS-KEY (cdi) OR TITLE-ABS-KEY (clostridioides AND difficile-induced AND colitis) OR TITLE-ABS-KEY (clostridium AND difficile-induced AND colitis) OR TITLE-ABS-KEY (clostridium AND difficile AND induced AND colitis) AND TITLE-ABS-KEY (mice) OR TITLE-ABS-KEY (mouse) AND TITLE-ABS-KEY (fecal AND microbiota AND transplantation) OR TITLE-ABS-KEY (fmt) AND TITLE-ABS-KEY (metabolic AND profiling) OR TITLE-ABS-KEY (metabolomics) OR TITLE-ABS-KEY (metabolites) OR TITLE-ABS-KEY (metabonomics) OR TITLE-ABS-KEY (lipidomics) OR TITLE-ABS-KEY (lipid AND profiling) OR TITLE-ABS-KEY (bile AND acids) OR TITLE-ABS-KEY (amino AND acids))

Google Scholar advanced search query:

('c. difficile' OR 'clostridioides difficile' OR 'cdi' OR 'clostridioides difficile-induced colitis' OR 'clostridium difficile-induced colitis') AND ('mice' OR 'mouse') AND ('fecal microbiota transplantation' OR 'fmt') AND ('metabolic profiling' OR 'metabolomics' OR 'metabolites' OR 'metabonomics' OR 'lipidomics' OR 'lipid profiling' OR 'bile acids' OR 'amino acids')
